# Prospective 12-month outcomes of combined iStent inject implantation and phacoemulsification in Asian eyes with normal tension glaucoma

**DOI:** 10.1186/s40662-022-00294-2

**Published:** 2022-07-05

**Authors:** Bryan Chin Hou Ang, Wenqi Chiew, Vivien Cherng Hui Yip, Chun Hau Chua, Wei Shan Han, Ivan O’Neill C. Tecson, Jeanne Joyce Ogle, Boon Ang Lim, Owen Kim Hee, Elton Lik Yong Tay, Vernon Khet Yau Yong, Hon Tym Wong, Leonard Wei Leon Yip

**Affiliations:** 1 National Healthcare Group Eye Institute, Tan Tock Seng Hospital, Singapore, Singapore; 2 National Healthcare Group Eye Institute, Woodlands Health Campus, Singapore, Singapore; 3 Yong Loo Lin School of Medicine, National University of Singapore, Singapore, Singapore; 4 Cardinal Santos Medical Centre, Manila, Philippines; 5 Hesed Eye Specialists, Singapore, Singapore

**Keywords:** iStent inject, Normal tension glaucoma, Minimally invasive glaucoma surgery, Asian

## Abstract

**Background:**

Glaucoma is the leading cause of irreversible blindness. Normal tension glaucoma (NTG) is a subset of open-angle glaucoma, demonstrating glaucomatous optic nerve damage in the absence of raised intraocular pressure (IOP). NTG is more prevalent in Asian populations. While generally slow-progressing, NTG may be associated with significant central visual field loss. In recent years, minimally invasive glaucoma surgery has been added to the armamentarium of glaucoma surgery. This prospective study aims to evaluate 12-month surgical outcomes of combined iStent inject (Glaukos Corporation, Laguna Hills, CA) implantation and phacoemulsification in Asian eyes with NTG.

**Methods:**

This is a prospective, single-centre case series of 30 eyes followed up until 12 months after surgery. Outcome measures included IOP, number of glaucoma medications, best-corrected visual acuity (BCVA) and intra and postoperative complications.

**Results:**

Mean age of subjects was 73.1 ± 6.3 years. Majority were ethnic Chinese (n = 27, 90%). Baseline medicated mean IOP was 13.8 ± 2.4 mmHg and mean number of glaucoma medications was 1.3 ± 0.7. Mean Humphrey visual field mean deviation was − 13.7 ± 7.6. The mean IOP reduction at all timepoints from postoperative month (POM) 3 onwards was statistically significant (all *P* < 0.05), with mean reduction of 1.2 mmHg (95% CI: 0.1–2.2, *P* = 0.037) by POM12. There was statistically significant reduction in mean number of medications from postoperative day (POD) 1 onwards (all *P* < 0.05), with mean decrease of 1.0 medication (95% CI: 0.9–1.1, *P* < 0.001) by POM12. By POM12, 25 (83.3%) eyes were medication-free. Three (10%) eyes had stent occlusion by iris requiring laser iridoplasty. One eye had gross hyphema which resolved on conservative management before POM1. Mean BCVA improved from the baseline 0.3 ± 0.3 logMAR to 0.1 ± 0.1 logMAR postoperatively (*P* < 0.001). There were no major adverse or sight-threatening events. No eyes required further glaucoma surgery during the 12-month follow-up period.

**Conclusion:**

Asian eyes with NTG which underwent combined iStent inject implantation and phacoemulsification demonstrated a significant and sustained reduction in IOP and glaucoma medications, up to 12 months postoperatively.

## Background

Glaucoma is the leading cause of irreversible blindness and in 2020, has been reported to cause blindness in 3.61 million people worldwide [1]. Normal tension glaucoma (NTG) is a subset of open-angle glaucoma (OAG), demonstrating glaucomatous optic nerve damage and visual field defects in the absence of raised intraocular pressure (IOP) [2]. Although usually slow-progressing, NTG is associated with debilitating central visual field loss [3]. Population studies have shown that NTG affects between 30–40% of patients with OAG in the United States, Netherlands and Italy [4–6], with a higher prevalence of between 47–92% found among Asian populations [2, 7].

Despite the low baseline IOPs in NTG, IOP reduction remains the mainstay of treatment to reduce disease progression [8]. Inadequate IOP reduction [9, 10] and IOP fluctuation [11] have been demonstrated to be risk factors of NTG progression. While first-line therapy usually involves the use of topical eyedrops, medications have been associated with side effects as well as high non-compliance rates of up to 50% [12, 13]. Surgery is indicated if there is disease progression despite maximal medical therapy. Traditional filtration surgeries, such as trabeculectomy surgery, have demonstrated good efficacy in eyes with NTG [8, 14, 15]. However, surgical risks are potentially sight-threatening and may include hypotony, choroidal detachment, blebitis, endophthalmitis, as well as high long-term failure rates [16, 17]. The risk of postoperative hypotony and hypotonus maculopathy, in particular, have been shown to be greater in NTG eyes compared to OAG eyes [18, 19].

More recently, minimally invasive glaucoma surgery (MIGS) has emerged as a viable alternative in glaucoma management. Encompassing a range of devices and techniques, MIGS reduces IOP through a variety of mechanisms. The iStent inject (Glaukos Corporation, Laguna Hills, CA) is an angle-based MIGS, a second-generation titanium trabecular meshwork bypass device that enhances physiologic aqueous outflow through the trabecular meshwork of the eye. It is used most in conjunction with cataract surgery in the treatment of mild to moderate OAG, having demonstrated good efficacy and safety profile across various studies [20, 21]. However, there is limited data on the use of the iStent inject in NTG [22, 23]. This study aims to evaluate 12-month surgical outcomes of combined iStent inject implantation and phacoemulsification in Asian eyes with NTG.

## Methods

### Study design

This was a prospective, interventional, non-washout case series conducted in a single tertiary ophthalmology centre in Asia. All surgeries were performed between June to November 2019. The inclusion criteria were: age ≥ 21 years old; presence of a visually-significant cataract; a clinical diagnosis of NTG accompanied by perimetric glaucomatous optic neuropathy [defined as having a cup-disc ratio (CDR) of ≥ 0.7 or CDR asymmetry of ≥ 0.2 between both eyes, accompanied by a visual field defect demonstrated on a 24–2 SITA-fast/SITA-standard visual field test]; open angle on gonioscopy (defined by < 2 quadrants of irido-trabecular contact on non-indentational gonioscopy); a nasal quadrant with Shaffer grade ≥ 3 on non-indentational gonioscopy, without peripheral anterior synechia, rubeosis or other angle abnormalities that could impair proper placement of the iStent inject device; and eyes on ≥ 1 glaucoma topical medication. Eyes with a history of glaucoma laser treatment or any other intraocular surgery were excluded from study recruitment.

All subjects underwent a preoperative standardized baseline assessment including IOP measurement with Goldmann applanation tonometry and slit-lamp biomicroscopy examination with gonioscopy, performed by a glaucoma fellowship-trained consultant ophthalmologist. Data from the latest Humphrey visual field (HVF) (Zeiss, Oberkochen, Germany) test, clinical refraction with best-corrected visual acuity (BCVA) results and stereodisc photos performed within 6 months before surgery were collected. Disease severity was categorized as early [HVF mean deviation (MD) ≤ 6 dB], moderate (6 dB < HVF MD ≤ 12 dB) and severe (HVF MD > 12 dB) using the Hodapp-Anderson-Parrish visual field criteria [24]. The decision for surgery, as well as the surgery itself, was performed by a glaucoma fellowship-trained consultant ophthalmologist.

The design of the study followed the tenets of the Declaration of Helsinki and ethics approval was obtained from the institution’s ethics review committee, the National Healthcare Group Domain Specific Review Board (NHG DSRB 2019/00134). Informed consent was obtained from all subjects.

### Surgical technique and postoperative care

All surgeries in this study were performed by fellowship-trained glaucoma consultant ophthalmologists who were accredited to perform iStent inject implantation and had performed at least 10 successful combined phacoemulsification and iStent inject implantation surgeries prior to commencement of the study.

Surgical steps were standardized across all surgeries in this study and were as follows. Phacoemulsification with intraocular lens insertion was first performed. After injection of viscoelastic to deepen the anterior chamber, intraoperative gonioscopy was performed to ensure the presence of an open nasal angle suitable for implantation of the iStent inject device. Thereafter, the iStent inject injector was inserted through the main corneal incision and two iStent inject devices were implanted into the trabecular meshwork, at least two clock hours apart. The viscoelastic was then removed and the corneal wounds sealed by hydration. Intracameral antibiotics were administered for all eyes, except in subjects with specific drug allergies.

The postoperative regimen for all subjects consisted of topical Tobradex (tobramycin and dexamethasone ophthalmic suspension) eyedrops applied 3-hourly for one week, followed by 6-hourly for 3 weeks. The decision regarding the continuation or stopping of preoperative glaucoma medications immediately after surgery was made at the discretion of the attending surgeon. Further escalation of glaucoma eyedrop medications, as well as decisions on further laser or surgical treatments were at the discretion of the attending surgeon. All postoperative clinical assessments and decision-making were performed by fellowship-trained glaucoma consultant ophthalmologists throughout the follow-up period.

### Outcome measures

Postoperative clinical visits were scheduled at postoperative day (POD) 1, postoperative week (POW) 1, postoperative month (POM) 1, POM3, POM6 and POM12. Gonioscopy was performed at all visits from POM1 onwards. Efficacy outcomes included change in IOP and anti-glaucoma medications from preoperative baseline. Safety outcome measures included intra and postoperative complications and change in BCVA after surgery. Progression was measured using changes in HVF parameters and CDR at POM12, compared to baseline.

Definitions of complete success, qualified success and failure were adapted from the World Glaucoma Association Guidelines [25]. Surgical “failure” was defined as one or more of the following:

1. IOP > 18 mmHg, or less than 20% reduction from baseline on two consecutive follow-up visits, from (and inclusive of) the 1-month postoperative timepoint, onwards;
2. IOP < 5 mmHg on two consecutive follow-up visits, from (and inclusive of) the 1-month postoperative timepoint, onwards;
3. Re-operations for glaucoma;
4. Loss of light perception after surgery or vision-threatening severe complications.

In view of our subjects having NTG with low baseline IOPs, we re-analysed our success outcomes using IOP thresholds of ≤ 15 mmHg and ≤ 12 mmHg, in addition to the threshold of ≤ 18 mmHg. Complete success was defined as achieving the IOP threshold without the use of anti-glaucoma medications. Qualified success was defined as achieving the IOP threshold with the use of anti-glaucoma medications. Only postoperative data from POM1 onwards was included for analysis of surgical success outcomes, in view of the IOP fluctuations sometimes observed within the first postoperative month due to transient factors such as retained viscoelastic.

The mean absolute error (MAE), defined as the difference between the postoperative and target spherical equivalent (SE), was calculated to reflect refractive outcomes.

### Statistical analysis

Standard statistical analysis was performed using IBM SPSS Statistics (version 27, IBM Corp, New York, USA). Continuous variables were described with mean standard deviation (SD), mean 95% confidence interval (95% CI), median interquartile range (IQR) or range, while categorical variables were expressed as frequencies (n) and percentages (%). Data distribution was evaluated for normality using Shapiro-Wilk test. Unpaired t-test or Mann–Whitney *U* test was used to compare continuous parameters before and after surgery, where appropriate. Comparisons of pre and postoperative proportions were performed using the Fisher’s Exact test. Efficacy outcomes were assessed by comparing the postoperative IOP and number of medications at each timepoint against preoperative data, using the Wilcox signed-rank test. A *P* value of less than 0.05 was considered statistically significant.

Sample size calculation for this prospective study was performed with reference from a previous pilot study conducted at our institution [26]. Preliminary results of the first 26 eyes that underwent combined phacoemulsification and iStent inject implantation in our institution showed favourable outcomes, with a statistically significant mean reduction in IOP of 2.2 mmHg from baseline. Assuming a standard deviation of 3.5 mmHg, a sample of 22 was required to detect a 2.2 mmHg mean reduction, at 0.8 power and 0.05 significance level. 40% (8) further subjects were included to compensate for dropouts, resulting in the final sample size of 30 subjects.

## Results

A total of 30 eyes from 30 subjects were analysed, with all subjects successfully completing follow-up until 12 months after the surgery. The average age of subjects was 73.1 ± 6.3 years and majority were ethnic Chinese (n = 27, 90%). Preoperatively, baseline medicated mean IOP was 13.8 ± 2.4 mmHg and subjects were on a mean of 1.3 ± 0.7 glaucoma medications. All 30 eyes were on at least one medication preoperatively. The mean HVF MD was − 13.7 ± 7.6 dB. Seventeen (56.7%) eyes had severe NTG, 8 (26.7%) eyes had moderate NTG and 5 (16.7%) eyes had early NTG. Demographics and baseline characteristics are summarized in Table 1.

**Table 1:** Demographics and baseline characteristics

Characteristics	NTG phaco-iStent (n = 30)
Age, mean (SD), years	73.1 (6.3)
Gender	
Male (%)	14 (46.7)
Female (%)	16 (53.3)
Race	
Chinese (%)	27 (90.0)
Indian (%)	3 (10.0)
Laterality of eye	
Right (%)	19 (63.3)
Left (%)	11 (36.7)
BCVA (logMAR), Median (IQR)	0.3 (0.2 – 0.4)
Number of anti-glaucoma medications	
Mean (SD)	1.3 (0.7)
Median (IQR)	1 (1 – 1)
Prior glaucoma surgeries	
No (%)	30 (100)
Yes (%)	0 (0)
CDR, median (IQR)	0.80 (0.70 – 0.90)
HVF MD, mean (SD)	− 13.7 (7.6)
HVF PSD, mean (SD)	7.4 (2.9)
CCT (μm), mean (SD)	540.5 (35.0)
IOP	
Mean (SD)	13.8 (2.4)
Median (IQR)	13.0 (12.0 – 16.0)

*NTG* = normal tension glaucoma; *SD* = standard deviation; *BCVA* = best-corrected visual acuity; *logMAR* = logarithm of the minimum angle of resolution; *IQR* = interquartile range; *CDR* = cup-disc ratio; *HVF* = Humphrey visual field; *MD* = mean deviation; *PSD* = pattern standard deviation; *CCT* = central corneal thickness; *IOP* = intraocular pressure

### Efficacy

**Fig. 1:**
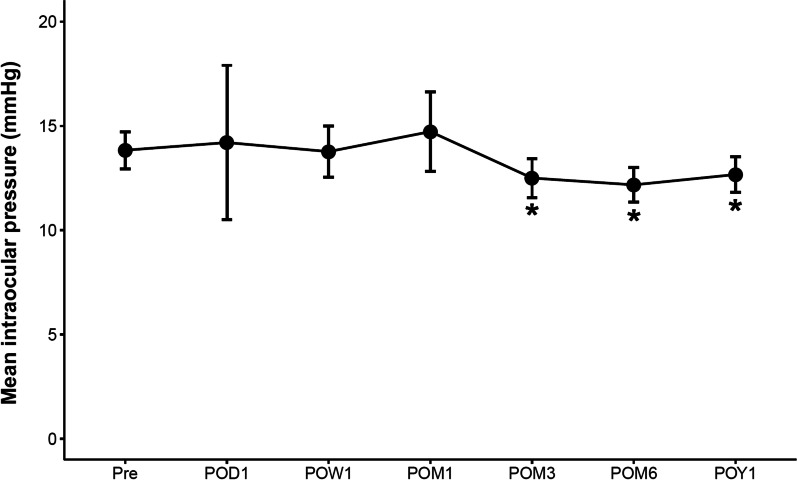
Postoperative change in mean intraocular pressure. *Denotes statistical significance at *P* < 0.05 and the error bars represent 95% confidence intervals. Pre, preoperative; POD, postoperative day; POW, postoperative week; POM, postoperative month; POY, postoperative year

**Fig. 2:**
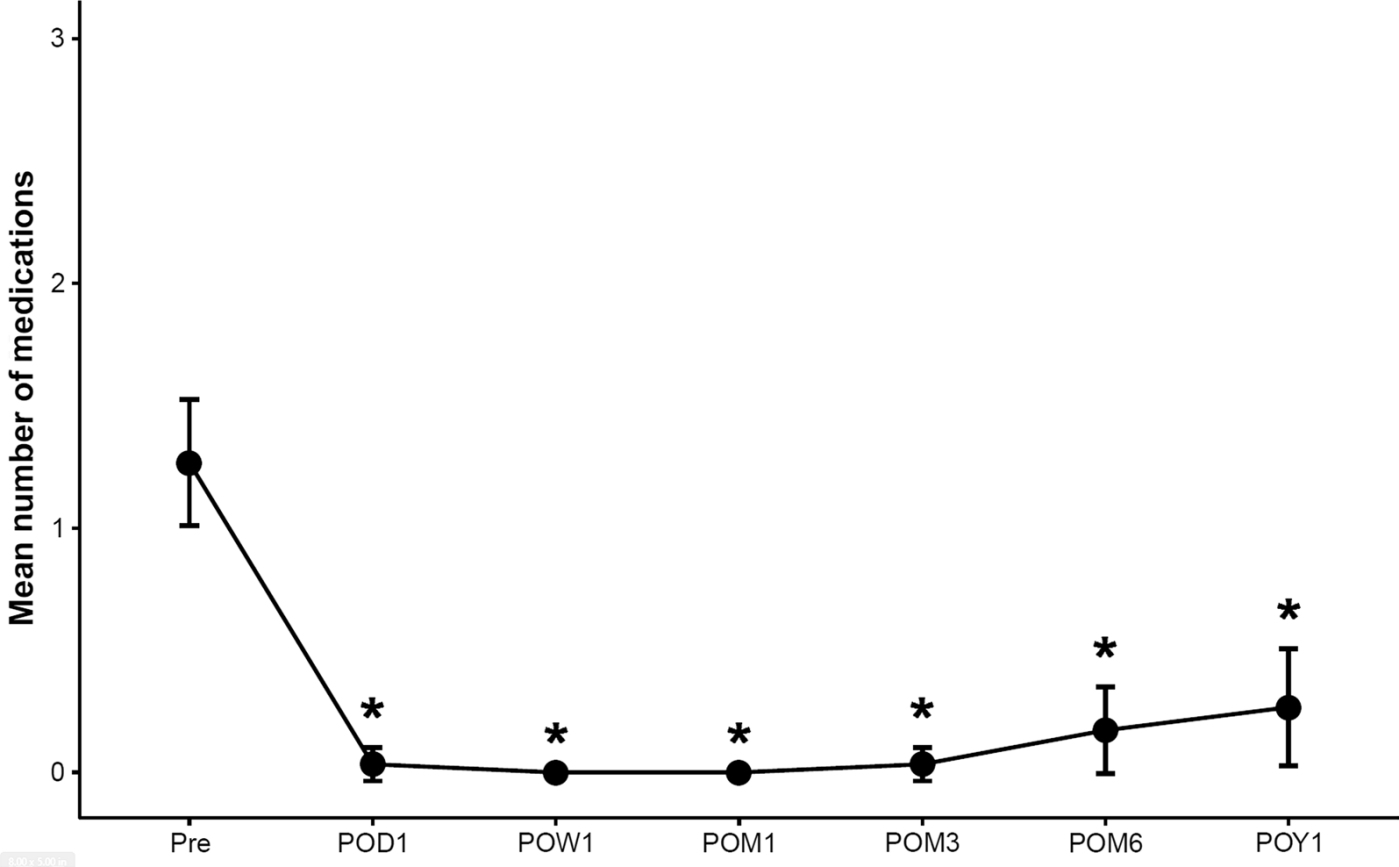
Postoperative change in mean number of anti-glaucoma medications. *Denotes statistical significance at *P* < 0.05, and the error bars represent 95% confidence intervals. Pre, preoperative; POD =, postoperative day; POW =, postoperative week; POM, postoperative month; POY, postoperative year

There was a statistically significant reduction in IOP from POM3 onwards (all *P* < 0.05) compared to baseline, with IOP decreasing by 1.2 mmHg (95% CI: 0.1 to 2.2, *P* = 0.037) by POM12 (Table 2 and Fig. 1). Concurrently, there was a statistically significant reduction in the mean number of medications compared to baseline from POD1 onwards (all *P* < 0.05), with a mean decrease of 1.0 medication (95% CI: 0.9 to 1.1, *P* < 0.001) by POM12 (Table 3 and Fig. 2). By POM12, 25 (83.3%) eyes were medication-free. Two (6.7%) eyes required one glaucoma medication and 3 (10%) eyes required two glaucoma medication. All 5 (16.7%) eyes requiring glaucoma medications at POM12 were eyes with severe NTG. None of the patients had an increase in the number of glaucoma medications postoperatively.

**Table 2:** Mean IOP at baseline and at each postoperative timepoint

Timepoint	No. of eyes present	IOP mean (95% CI)	Mean (95% CI) differences from baseline	*P* value* (pre *vs*. post)
Pre-op	30	13.8 (12.9 – 14.7)		
Day 1 post-op	30	14.2 (10.5 – 17.9)	0.3 (− 3.0 – 3.8)	0.263
Week 1 post-op	30	13.8 (12.5 – 15.0)	− 0.1 (− 1.1 – 1.0)	0.765
Month 1 post-op	29	14.7 (12.8 – 16.6)	0.8 (− 1.0 – 2.6)	0.881
Month 3 post-op	30	12.5 (11.6 – 13.4)	− 1.3 (− 2.5 – − 0.2)	0.023
Month 6 post-op	29	12.2 (11.4 – 13.0)	− 1.7 (− 2.7 – − 0.7)	0.002
Year 1 post-op	30	12.7 (11.8 – 13.5)	− 1.2 (− 2.2 – − 0.1)	0.037

*IOP* = intraocular pressure; *CI* = confidence interval; *Pre-op* = preoperative; *Post-op* = postoperative

^*^Wilcox signed-rank test

**Table 3:** Mean number of medications at baseline and at each postoperative timepoint

Timepoint	No. of eyes present	No. of medications mean (95% CI)	Mean (95% CI) differences from baseline	*P* value* (pre *vs.* post)
Pre-op	30	1.3 (1.0 – 1.5)		
Day 1 post-op	30	0.0 (0.0 – 0.1)	− 1.2 (− 1.5 – − 1.0)	< 0.001
Week 1 post-op	30	0.0 (0.0 – 0.0)	− 1.3 (− 1.5 – − 1.0)	< 0.001
Month 1 post-op	29	0.0 (0.0 – 0.0)	− 1.3 (− 1.5 – − 1.0)	< 0.001
Month 3 post-op	30	0.0 (0.0 – 0.1)	− 1.2 (− 1.5 – − 1.0)	< 0.001
Month 6 post-op	29	0.2 (0.0 – 0.4)	− 1.1 (− 1.3 – − 0.9)	< 0.001
Year 1 post-op	30	0.3 (0.0 – 0.5)	− 1.0 (− 1.1 – − 0.9)	< 0.001

*IOP* = intraocular pressure; *CI* = confidence interval; *Pre-op* = preoperative; *Post-op* = postoperative

*Wilcox signed-rank test

### Safety

Intraoperatively, over-implantation of at least one of the pair of iStent inject devices occurred in 3 eyes (10%). In these cases, a second iStent inject delivery system was utilized and eventually, a total of 2 stents remained visible in each of these eyes. In one (3.3%) eye with failed implantation using the first iStent inject delivery system, a second delivery system was utilized, with a total of 3 stents visible at the end of surgery. Intra-operative zonulysis occurred in one eye, however, this was unrelated to the implantation of the iStent inject. Postoperatively, 3 (10%) eyes had stent occlusion by iris requiring laser iridoplasty. One (3.3%) eye had gross hyphema which resolved on conservative treatment before POM1. Prior to surgery, this subject was on single anti-platelet treatment, but this was withheld a week before surgery. Episodes of high IOP (defined as > 21 mmHg) occurred in 2 (6.7%) eyes at the POM1 timepoint. Cystoid macula oedema occurred in 2 (6.7%) eyes, which resolved with topical steroids and non-steroidal anti-inflammatory eyedrops. No major adverse or sight-threatening events occurred and none of the eyes required further glaucoma surgery throughout the follow-up period.

Mean BCVA improved from a baseline logMAR of 0.3 ± 0.3 to 0.1 ± 0.1 at POM12 (*P* < 0.001) (Table 4). There was no evidence of disease progression up to the POM12 timepoint (Table 5), with no statistically significant change in the CDR (*P* = 0.450), HVF MD (*P* = 0.636) and HVF pattern standard deviation (PSD) (*P* = 0.364).

**Table 4:** Mean and median values of BCVA at pre-op and POM12

	Pre-op	POM12	*P* value
BCVA (logMAR)			
Mean ± SD	0.3 ± 0.3	0.1 ± 0.1	< 0.001*
Median (IQR)	0.3 (0.2 – 0.4)	0.1 (0.0 – 0.2)	

*BCVA* = best-corrected visual acuity; *logMAR* = logarithm of the minimum angle of resolution; *POM12* = postoperative month 12; *SD* = standard deviation;  *IQR* = interquartile range

^*^Wilcoxon signed-rank test

**Table 5:** Mean and median values of HVF MD, HVF PSD, CDR at pre-op and POM12

Parameters		Pre-op	POM12	*P* value
HVF (MD)				
Mean (95% CI)		− 13.7 (− 16.5 – − 10.8)	− 13.3 (− 16.5 – − 10.0)	0.636^
Median (IQR)		− 12.7 (− 18.1 – − 8.1)	− 10.9 (− 20.4 – − 5.4)	
HVF (PSD)				
Mean (95% CI)		7.4 (6.4 – 8.5)	7.8 (6.5 – 9.2)	0.364^
Median (IQR)		7.4 (5.4 – 9.4)	7.9 (4.9 – 10.8)	
CDR				
Mean ± SD		0.8 ± 0.1	0.8 ± 0.1	0.450*
Median (IQR)		0.80 (0.70 – 0.90)	0.80 (0.70 – 0.90)	

*HVF* = Humphrey visual field; *MD* = mean deviation; *PSD* = pattern standard deviation; *CDR* = cup-disc ratio; *pre-op* = preoperative; *POM12* = postoperative month 12; *CI* = confidence interval; *IQR* = interquartile range; *SD* = standard deviation

^*^Wilcoxon signed-rank test

^Paired t-test

The MAE was − 0.13 ± 0.08 D (n = 29, one eye had missing refraction data at POM1 and was excluded from analysis). 82.8% (24 out of 29) of all eyes achieved a postoperative refraction within 0.5 D of target, while 96.6% (28 out of 29) of all eyes achieved a postoperative refraction within 1.0 D of target.

**Fig. 3:**
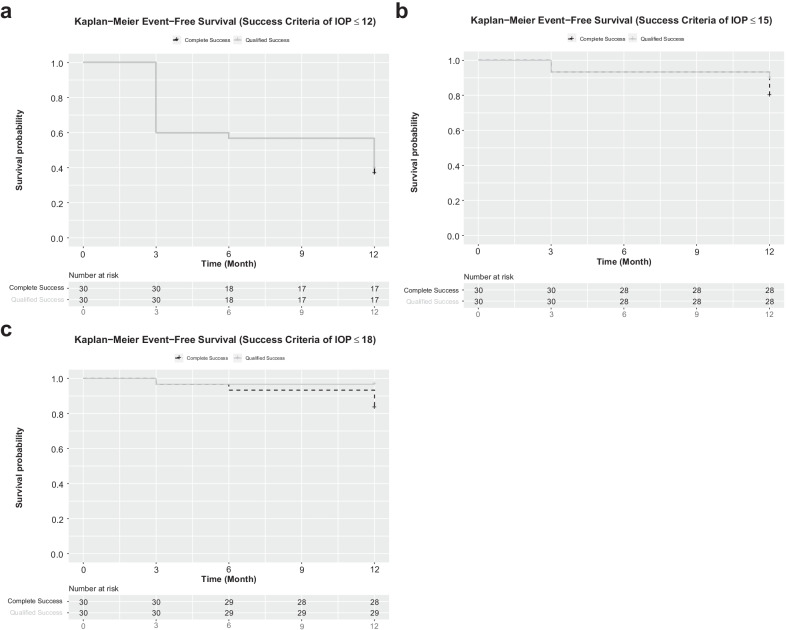
Kaplan-Meier (KM) plots showing cumulative probabilities of complete and qualified success for success criteria of (**a**) IOP ≤ 12 mmHg, (**b**) IOP ≤ 15 mmHg and (**c**) IOP ≤ 18 mmHg, with number of eyes at risk indicated in panels below respective KM plots

Kaplan-Meier survival analysis was used to demonstrate the cumulative probability of success, as defined by one of three IOP-threshold criteria (≤ 12, ≤ 15 and ≤ 18 mmHg) at 12 months of follow-up (Fig. 3).

## Discussion

The safety and efficacy of iStent inject implantation in the treatment of OAG has been well established in Caucasian eyes with OAG, when performed as a standalone procedure [27] as well as in combination with cataract surgery [28]. In contrast, few studies have explored iStent inject implantation in NTG eyes [22, 23], with no prospective study published to date reporting outcomes particularly in Asian eyes, despite the well-known higher prevalence of NTG in Asian populations [29]. The efficacy of iStent inject in NTG remains unclear and unlike in primary open-angle glaucoma (POAG), may be limited by the already low baseline IOP [29] and the even lower, at times single-digit, target IOPs required to reduce disease progression [15, 30]. The efficacy of angle-based MIGS is also understood to be limited by distal aqueous outflow pathway resistance and the episcleral venous pressure of the eye [20]. This limitation in efficacy may be more clinically relevant in eyes with NTG.

Our study has successfully demonstrated that combined cataract surgery and iStent inject implantation does result in a sustained and statistically significant mean IOP reduction of 1.2 mmHg up to 12 months postoperatively. Salimi et al. [23], in a retrospective study on 62 eyes with mostly mild to moderate NTG, reported a mean IOP reduction of 3.5 mmHg at POM12 from a baseline IOP of 15.82 ± 2.9 mmHg. A subanalysis of the outcomes in the NTG eyes in another study by Neuhann et al. [31] showed a mean IOP reduction of 3.6 mmHg at POM12, from a baseline IOP of 17.1 mmHg. Rho et al. [32] described a mean IOP reduction of 2.6 mmHg from 15.1 ± 2.9 mmHg to 12.5 ± 2.0 mmHg at POM6 in 36 Korean eyes. The higher IOP reduction observed in these studies compared to ours may be in part due to the higher baseline IOPs in these studies, compared to the lower baseline IOP of 13.8 ± 2.4 mmHg in our study. Similarly, across studies on NTG eyes, the mean IOP reduction after iStent inject implantation appears to be lower compared to that in OAG eyes [27, 28, 33], likely also due to higher baseline IOPs in OAG eyes [29].

Nonetheless, the magnitude of IOP reduction demonstrated in our study after combined surgery appears greater than that after standalone cataract extraction in NTG eyes. Standalone phacoemulsification may decrease IOP by altering the mechanisms of aqueous humour outflow [34]. Several theories involving the decrease in IOP after lens removal have been postulated, such as decrease in aqueous humour production due to contraction of the lens capsule resulting in vitreous pull by the ciliary band fibers [35], as well as improvement of outflow of aqueous humour via uveoscleral outflow [36, 37] or via the trabecular and Schlemm’s canal [38]. One study evaluating the effect of standalone cataract surgery on IOP in Korean eyes at 1–3 years of follow-up reported a mean IOP reduction of 0.78 mmHg [39]. Another similar study in NTG eyes showed a mean IOP reduction of 1.7 mmHg at POM1 with no reduction in glaucoma medications after standalone cataract surgery [40]. Our study, in contrast, found a statistically significant mean IOP reduction of 1.2 mmHg and reduction in at least 1 glaucoma medication up to the POM12 timepoint. Other head-to-head studies have also demonstrated the superior IOP-lowering efficacy of combined surgery with iStent implantation over standalone phacoemulsification surgery [28, 34]. Samuelson et al. [28] reported that at 24 months postoperatively, 75.8% of eyes (n = 387) who underwent combined iStent and phacoemulsification surgery experienced ≥ 20% reduction from baseline unmedicated IOP, compared to 61.9% of eyes (n = 118) who underwent standalone phacoemulsification surgery (*P* = 0.005).

Beyond IOP reduction, a significant proportion of eyes (n = 25, 83.3%) in our study remained medication-free at 12 months after surgery. This statistic is similar to that from another study [32] in Korean eyes which reported that 83.3% of the 36 patients who underwent combined iStent inject implantation and phacoemulsification were medication-free at 6 months. The benefits of reducing the medication burden of glaucoma patients have been well documented. These include the avoidance of medication related side-effects, non-compliance, non-adherence and long-term costs [11–13], with significant improvement in the quality of life of patients [41]. The 5 (16.7%) eyes requiring glaucoma medications at POM12 all had severe NTG and given the advanced stage of disease, it may be possible that there was a lower threshold for the attending ophthalmologist to restart medications. It is unlikely that the need to restart medications in these eyes was related to stent patency as only one of these 5 eyes encountered prior stent occlusion and underwent successful laser iridoplasty. Other studies have demonstrated that patients with a higher preoperative medication burden are likely to have a lower chance of being medication-free postoperatively [42].

In terms of refractive outcomes, our study demonstrated minimal influence of iStent inject implantation on the MAE (− 0.13 ± 0.08 D), with 82.8% of eyes achieving a postoperative refraction within 0.5 D of target. These outcomes remain well within benchmark standards for refractive outcomes after cataract surgery [43]. Prior literature has also demonstrated that trabecular bypass stents are unlikely to affect refractive outcomes [32, 44], in contrast to other suprachoroidal MIGS devices, which have been shown to be associated with myopic shifts [45].

The safety profile of the iStent inject has been well established [27, 28, 46]. The low incidence of postoperative hypotony (0–2.6%) [27, 28, 33, 47] is likely due to the limiting presence of the episcleral venous pressure floor. This is in contrast to filtering surgery such as trabeculectomy, while effective in the treatment of NTG, has a significant risk of hypotony (up to 28%) [18, 19] and bleb-related complications [15, 17, 18]. The excellent safety profile of combined cataract surgery and iStent inject in our study is consistent with findings from other larger OAG studies [27, 28, 33]. Complications were uncommon and mostly self-limiting, with no sight-threatening events, endophthalmitis or hypotony. Notably, 3 (10%) eyes encountered occlusion of at least one of the pair of stents. The occlusion for all 3 eyes were observed at POM3 or earlier and all underwent laser iridoplasty, with all the stents remaining patent after treatment. Two of the 3 eyes had subsequently lower IOPs at the following postoperative timepoints and both remained medication-free. Stent occlusion was not reported in the study by Salimi et al. [23] and was reported to occur only rarely (0–4%) in other studies in OAG eyes [27, 28, 33]. The slightly higher incidence of stent occlusion in our study may be related to the narrower angles, shorter anterior chamber depths [48] and a more anterior insertion of the iris [49] in Asian eyes. However, these parameters were not examined in this study.

This study has several limitations. Firstly, as a non-comparative, single-arm case series with no phacoemulsification-alone control group, this study could not quantitatively assess the additional effect of the iStent inject over cataract extraction alone in NTG eyes. Other studies examining the IOP-lowering effect of phacoemulsification alone in NTG, however, may be used as a surrogate for data comparison [40, 50, 51]. Secondly, this was a non-washout study and does not reflect the true IOP-lowering efficacy of the surgery without removing the confounding factors of medication compliance and effect. Nonetheless, non-washout studies may better represent real-world clinical experience. Thirdly, there was no standardized protocol to guide postoperative treatment decisions, including the escalation of medications and laser iridoplasty treatment for stent occlusion, hence we could not control for individual surgeon preference and thresholds for treatment. Lastly, a longer study follow up duration beyond 12 months would be preferable for detecting NTG progression, with NTG understood to be a slow-progressing disease [52]. Longer-term studies may also demonstrate changes in efficacy, as some have demonstrated a decrease in efficacy following combined iStent inject implantation with phacoemulsification in POAG, beyond 12 months [31, 53].

Despite the above, this study is, to the best of our knowledge, the first prospective study to report outcomes of combined cataract surgery and iStent inject implantation in Asian eyes with NTG. While the IOP-lowering effect in NTG appears to be more modest compared to that observed in POAG eyes, the reduction in medication burden is significant and is likely to improve the quality of life for patients. The impressive safety profile of the iStent inject demonstrated in this study is consistent with those in established literature. The role of the iStent inject in the NTG treatment may be better defined with longer-term, comparative randomized control trials, as well as cost-effectiveness and quality of life studies. Future development of preoperative imaging modalities to better assess aqueous outflow pathways and distal outflow resistance may allow more targeted patient selection and device placement, resulting in greater IOP-lowering efficacy [54], which will be of particular importance in NTG eyes with lower target pressures.

## Conclusion

Asian eyes with NTG which underwent combined iStent inject implantation and phacoemulsification demonstrated a significant and sustained reduction in both IOP and glaucoma medications, up to 12 months postoperatively. Coupled with a high safety profile, combined iStent inject implantation and phacoemulsification may be recommended for Asian patients with normal tension glaucoma.

## Data Availability

The datasets analysed during the current study is available from the corresponding author on reasonable request.

## Abbreviations

BCVA: Best-corrected visual acuity
CDR: Cup-disc ratio
CI: Confidence interval
HVF: Humphrey visual field
LogMAR: Logarithm of the minimum angle of resolution
MD: Mean deviation
MIGS: Minimally invasive glaucoma surgery
NTG: Normal tension glaucoma
IQR: Interquartile range
IOP: Intraocular pressure
OAG: Open-angle glaucoma
PSD: Pattern standard deviation
POAG: Primary open-angle glaucoma
POD: Postoperative day
POM: Postoperative month
POW: Postoperative week
SD: Standard deviation
